# Automated classification of mouse pup isolation syllables: from cluster analysis to an Excel-based “*mouse pup syllable classification calculator*”

**DOI:** 10.3389/fnbeh.2012.00089

**Published:** 2013-01-09

**Authors:** Jasmine M. S. Grimsley, Marie A. Gadziola, Jeffrey J. Wenstrup

**Affiliations:** ^1^Department of Anatomy and Neurobiology, Northeast Ohio Medical UniversityRootstown, OH, USA; ^2^School of Biomedical Sciences, Kent State UniversityKent, OH, USA

**Keywords:** cluster analysis, mouse pup calls, vocalization, isolation calls, mouse song, communication call

## Abstract

Mouse pups vocalize at high rates when they are cold or isolated from the nest. The proportions of each syllable type produced carry information about disease state and are being used as behavioral markers for the internal state of animals. Manual classifications of these vocalizations identified 10 syllable types based on their spectro-temporal features. However, manual classification of mouse syllables is time consuming and vulnerable to experimenter bias. This study uses an automated cluster analysis to identify acoustically distinct syllable types produced by CBA/CaJ mouse pups, and then compares the results to prior manual classification methods. The cluster analysis identified two syllable types, based on their frequency bands, that have continuous frequency-time structure, and two syllable types featuring abrupt frequency transitions. Although cluster analysis computed fewer syllable types than manual classification, the clusters represented well the probability distributions of the acoustic features within syllables. These probability distributions indicate that some of the manually classified syllable types are not statistically distinct. The characteristics of the four classified clusters were used to generate a Microsoft Excel-based mouse syllable classifier that rapidly categorizes syllables, with over a 90% match, into the syllable types determined by cluster analysis.

## Introduction

The isolation vocalizations of infant mice are used as behavioral markers of stress (Rupniak et al., 2000) or disease state (Ricceri et al., 2007; Scattoni et al., 2008; Young et al., 2010; Wohr et al., 2011a). While several studies show that the rate of vocalizations relates to stress, disease state, social context, or strain (Rupniak et al., 2000; Hahn and Schanz, 2002; Young et al., 2010), it is now clear that the types of emitted syllables are also affected in pups (Scattoni et al., 2008; Young et al., 2010; Chabout et al., 2012) and adults (Chabout et al., 2012). Identification of changes in these syllable categories may provide further insight into disease models, but current methods of manual categorization are slow and biased by the investigator. This study first uses cluster analysis to determine the number of discrete syllable categories produced by pups, then describes and evaluates a Microsoft Excel-based automated calculator to classify mouse pup isolation calls into these syllable types.

Generating filters within a spreadsheet to classify syllables is a relatively simple task; however, the determination of appropriate filter functions is more complex. How many discrete syllables do mice produce? Surprisingly, no study has attempted to determine the number of statistically distinct syllable types produced by mouse pups. Instead, the number of syllable categories has been determined subjectively by the experimenter, based on visual inspection of the spectrogram, with syllables sharing unique spectro-temporal features grouped together (Sales and Smith, 1978; Portfors, 2007; Sugimoto et al., 2011).

The current syllable categories for mouse pup vocalizations have evolved over the last 30+ years. Initially, isolation vocalizations were classified into one or more of five categories (Sales and Smith, 1978). These categories included syllables with sudden frequency steps and four other categories with varying rates of frequency modulation (FM). Arbitrary boundaries were set between slow FM, regular FM, and rapid FM categories. Branchi et al. (1998) morphed these categories (Sales and Smith, 1978) with those used for gerbil communication (Holman and Seale, 1991) to construct five categories; only the frequency stepped category of Sales and Smith (1978) remained. New arbitrary syllable borders were introduced; the rates of FM change used by Sales and Smith (1978) were replaced by measurements of the magnitude of the FM change (Branchi et al., 1998). A syllable was considered constant frequency if the FM changed <8 kHz in either the positive or negative direction, whereas a syllable with an FM change >8 kHz was classified as an FM syllable. More recently, Scattoni et al. (2008) proposed 10 categories that amalgamated the categories set out by Branchi et al. (1998) with observations from rat vocalizations (Brudzynski et al., 1999) and the categories identified for the analysis of adolescent mouse vocalizations (Panksepp et al., 2007). Scattoni and colleagues' new boundary to distinguish FM from constant frequency syllables was 6.25 kHz, as opposed to the 8 kHz FM boundary used previously (Branchi et al., 1998), no justification for the change was given. The FM syllables were further separated into up and down categories based on the direction of modulation. The FM boundary changed again to 6 kHz (Grimsley et al., 2011), without justification, and a further syllable category was added, increasing the count to 11 categories. The current study examines the probability distributions of the directional FM syllables to determine whether FM syllables are statistically distinct, and if so, where the appropriate boundaries should be set.

New technology allowing for the easy automatic extraction of the acoustic features of syllables makes the analysis of large data sets of communication calls feasible (Liu et al., 2003; Holy and Guo, 2005). Several techniques have been implemented for the automatic identification of syllable types in other species, including hidden Markov models (Ren et al., 2009), neural network models (Pozzi et al., 2010), and forms of cluster analysis (Takahashi et al., 2010). Hammerschmidt et al. (2012) used two-step cluster analysis to classify infant mouse vocalizations into two categories, short syllables and long syllables.

Both Markov models and neural network models rely on experimenter predetermination of the number of syllable categories prior to analysis. This process leads to experimenter bias. An alternate approach involves cluster analysis, a commonly used method for the identification of homogenous groups within data. Unlike other forms of cluster analysis, two-step cluster analyses do not require a priori knowledge of the number of syllable types in a data set. Instead, the experimenter identifies which variables to include in the clustering algorithm. The main bias of two-step cluster analysis comes from the variables the experimenter chooses to include.

Cluster analyses have been used to classify syllables in several species, including primates (Pozzi et al., 2010), owls (Nagy and Rockwell, 2012), and rats (Takahashi et al., 2010). For the analysis of primate vocalizations (Pozzi et al., 2010), several variables were used to cluster the calls into subtypes, including the frequency of the fundamental (*F*_0_), the frequency of the first three harmonics (measured at the start, middle, and end of each vocalization), and the duration. The coherence with manual classification was high at 88.4%, with 100% correspondence for six of the eight categories. In a recent study of rats, only two variables, peak frequency and duration, were used to determine the number of vocalization types (Takahashi et al., 2010). This analysis identified three clusters of vocalizations at three distinct frequency bands, but did not categorize subtypes within these bands. If more information were available to the cluster algorithm, more clusters may have been determined. In mouse, eight features of mouse pup vocalizations were used as input variables for two-step cluster analysis (Hammerschmidt et al., 2012). Surprisingly, these eight variables separated vocalizations into only two categories that are very different from manual classifications: one containing short vocalizations, and one containing both longer vocalizations and those with pitch jumps (Hammerschmidt et al., 2012).

We have previously characterized syllables of the CBA/CaJ mouse strain across development using standard manual categorical classification (Grimsley et al., 2011). Our aim here is to generate a tool for the automatic classification of mouse pup isolation calls into syllable categories. The first step is to determine, using an automated two-step clustering technique, the number of statistically distinct syllable types within a data set, and to compare these categories with those of experimenter-derived categories. Because published syllable categories utilize spectrographic analysis of the frequency contour for manual analysis (Portfors, 2007; Scattoni et al., 2008; Grimsley et al., 2011), our automated analysis is based on tracking the fundamental frequency over time. Input variables for the algorithm thus include the start, middle, and end frequency of the fundamental. Further, since mouse pup vocalizations include discontinuous frequency contours (Holy and Guo, 2005), an additional variable is the number of frequency steps. Although the automated analysis resulted in fewer syllable categories, the basis for the automated classification became clear when the probability distributions of the calls were examined. By exploring the probability distributions of derived clusters we were able to generate a Microsoft Excel spreadsheet that can automatically classify new data sets of mouse pup isolation syllables from CBA/CaJ mice and other strains rapidly and consistently (see Supplementary materials).

## Materials and methods

### Subjects

All procedures were approved by the Institutional Animal Care and Use Committee at the Northeast Ohio Medical University (Approval ID number 10-001). The CBA/CaJ mouse vocalization data set has been described previously (Grimsley et al., 2011). The present analysis was restricted to isolation vocalizations from animals aged p5, p7, p9, p11, and p13 (15 pups from three litters). Briefly, mouse pups were isolated within a sound proof booth and their vocalizations were recorded during a 5-min isolation period. Each mouse was placed on a fresh absorbent surface in a plastic chamber that was cleaned with 80% ethanol between each pup to eliminate odor cues (Keller et al., 2006; Wesson et al., 2008). The chamber was maintained at a temperature of 26 °C. Body weight was not recorded. Acoustic signals were recorded by an ultrasonic condenser microphone (Avisoft Bioacoustics) situated 5 cm above the recording chamber. The recording system was flat (±3 dB) between 20 kHz and 140 kHz, with a low frequency roll-off of 12 dB per octave; the microphone signal was digitized at 500 kHz and 16-bit depth. The same protocol was used to record a sample of isolation calls from three IRW (inbred Rocky Mountain White) mice from one litter at age p5, a strain used primarily for retrovirus infection studies, and from three C57BL/6 mice from one litter, the most widely used inbred strain, at age p7.

### Data analysis

Isolation calls were analyzed by SASLab Pro (version 5.2.05, Avisoft Bioacoustics). Features of syllables from CBA/CaJ mice used in the previous study (Grimsley et al., 2011) were retained to facilitate comparisons between the cluster analysis, the automated classification via the *mouse syllable classification calculator*, and the prior manual categorization. The start and end timestamps of the individual syllables within the isolation calls of IRW and C57BL/6 mice were detected using the single line threshold method within the automated parameter measurements of SASLab. Signals with amplitudes 10 dB above the noise floor were detected, with a 5 ms hold time to allow for short periods of low intensity within a syllable. The computed markers were checked visually and any markers surrounding acoustic signals relating to movement noise were removed.

Spectral measurements were computed from the spectrograms computed for each syllable (Hamming window, FFT length = 1024, frame size = 100%, overlap = 98.43%), with the frequency range restricted to 40–130 kHz. The frequency contour of each syllable was extracted using Automated Parameter Measurements, a feature of SASLab. The dominant frequency (held in the *F*_0_) of labeled syllables was automatically computed at nine evenly spaced time points; up to 25 time points were taken initially, but nine were found to be sufficient. These data, along with the syllable duration, start time and the mean dominant frequency were automatically saved. During low amplitude portions of a syllable, spurious *F*_0_ values tended to fall to the extremes of the spectrogram; thus, extracted *F*_0_ values were further bandpass filtered (range 41–129 kHz), as these spurious values would affect other calculations. When values fell outside this range, the frequency value was replaced with a value equal to the previous data point in the contour; this filter is included within the *mouse syllable classification calculator*. The nine-point frequency contour was used to determine the number of discontinuous frequency steps that occurred within a syllable. The criterion for these frequency steps was determined by examining the probability distribution of frequency transitions between adjacent points along the frequency contour. For calls at all ages and in all strains, we observed bimodal distributions in which larger frequency transitions indicated discontinuous frequency steps.

In a range of rodent phenotypes, pups from the same litter may be more similar than pups from different litters. To account for the potential bias in such litter effects, littermates are often considered to be non-independent observations (Zorrilla, 1997). To test whether litter effects were present in our data set, we performed a series of Two-Way nested ANOVAs (pup × litter) (McAllister et al., 2012) on several features of vocalizations, including duration, mean dominant frequency, and bandwidth. Within the nested ANOVAs, the subgroup pup was nested within the parent-group litter.

### Characterization of syllable types

Syllables from CBA/CaJ mice were used to determine the number of distinct syllable types. Syllables from p9 mice were excluded from this analysis so that they could be used as a novel data set to test the *mouse syllable classification calculator*. P9 syllables were chosen because they were the center point of the developmental range of the data set used in the study. Syllables were classified into distinct categories using two-step cluster analysis (SPSS, v.18). Unlike the majority of clustering algorithms, two-step cluster analysis (Chiu et al., 2001) is effective with large data sets. In the first step, the algorithm pre-clusters the data using a sequential processing method (Theodoridis and Koutroumbas, 1999). In the second step, it groups these pre-clusters into the automatically designated number of clusters using the agglomerative hierarchical clustering method (SPSS, 2001). A log-likelihood distance measure was chosen to determine the distance between syllable clusters, which is appropriate for the continuous variables used here. Default SPSS setting were used for both the maximum branches per leaf node (8), and maximum tree depth (3). The maximum leaf nodes determines the maximum number of “child” nodes a tree node can have in the hierarchical stage of the clustering and the maximum tree depth determines the number of levels of the tree; these would need to be altered from default for a small data set (less than ~1000 syllables). Schwarz's Bayesian Criterion was used to determine the number of distinct clusters. The silhouette of cohesion was used to determine the quality and separation of clusters (Tan et al., 2006); a value greater than 0.5 indicates highly separable clusters (Elleithy, 2010; Mooi and Sarstedt, 2011). Because two-step cluster analyses can be confounded by variables with correlations greater than 0.9, two-tailed bivariate correlations were computed to test for any correlations greater than *r* = 0.9 (Mooi and Sarstedt, 2011).

## Results

### Two-step cluster analysis

A total of 15,116 CBA/CaJ mouse pup syllables were used to determine the number of distinct syllables within the repertoire (p5, 3145; p7, 4329; p11, 4560; p13, 3082). The majority of animals emitted vocalizations at each age (mean call number: p5, 201; p7, 309; p11, 450; p13, 326).

**Figure 1:**
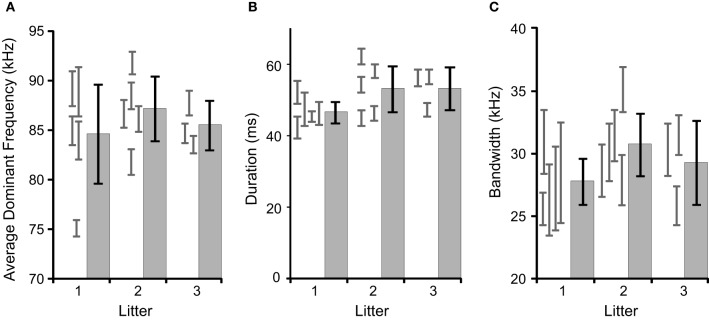
**Comparisons of inter- and intra-litter differences in basic acoustic features.** The gray histograms with black error bars represent the mean and 95% confidence intervals for litters, with the gray error bars representing 95% confidence intervals for the individual pups contributing to the litter. There were no significant inter-litter effects. **(A)** The average dominant frequency, **(B)** average syllable duration, **(C)** average syllable bandwidth.

We tested for litter effects by comparing several acoustic features between pups and litters at age p11, as it is later in development (see Figure 1). The results of a mixed-model nested ANOVA are that there is significant variation among pups within litters [duration: *F*_(10, 4547)_ = 22.7, *p* < 0.001; mean dominant frequency: *F*_(10, 4547)_ = 45.7, *p* < 0.001; bandwidth: *F*_(10, 4547)_ = 7.9, *p* < 0.001] and not significant variation among litters [duration: *F*_(2, 10)_ = 2.5, *p* = 0.133; mean dominant frequency: *F*_(2, 10)_ = 1.1, *p* = 0.363; bandwidth: *F*_(2, 10)_ = 1.0, *p* = 0.407]. We also conducted these analyses on the data set pooled across ages and found no litter effects (data not shown). Individual pups were therefore treated as independent observations.

**Figure 2:**
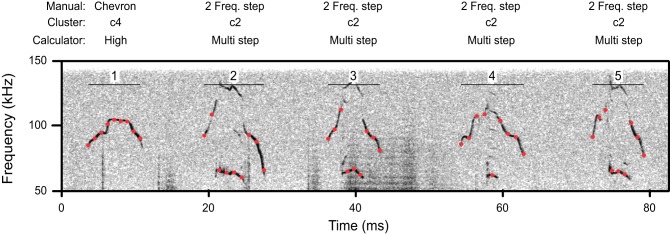
**A spectrogram illustrating a segment of syllables from a p11 CBA/CaJ pup.** *Red dots* indicate the location of automatic measurements of the nine-point frequency contour. *Above*, the classification of these syllables is shown by manual classification, cluster analysis, and the mouse call calculator. Syllables 2–5 have frequency steps.

**Figure 3:**
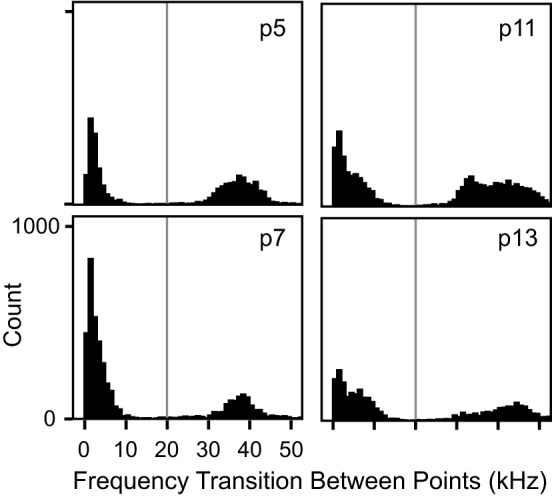
**Histograms of transitional frequency modulation at each age.** There was a bimodal distribution of frequency transitions at each age (*b* > 0.55 in all instances). Any frequency transition greater than 20 kHz, marked by the *vertical gray line*, was considered to represent a spectro-temporal discontinuity, i.e., a frequency step.

For each syllable, the automated nine-point fundamental frequency contour was first computed. Figure 2 shows this analysis as red dots superimposed onto the spectrograms of several syllables. Next, the distribution of frequency transitions was analyzed to develop a criterion for discontinuous frequency steps. This analysis showed a significantly bimodal distribution at each age (coefficient of bimodality, *b* > 0.55) (Figure 3). We chose 20 kHz as the criterion marking discontinuous frequency steps, as it falls roughly midway between the peaks of the distributions at each age. Figure 2 shows several examples of syllables with these frequency steps.

**Figure 4:**
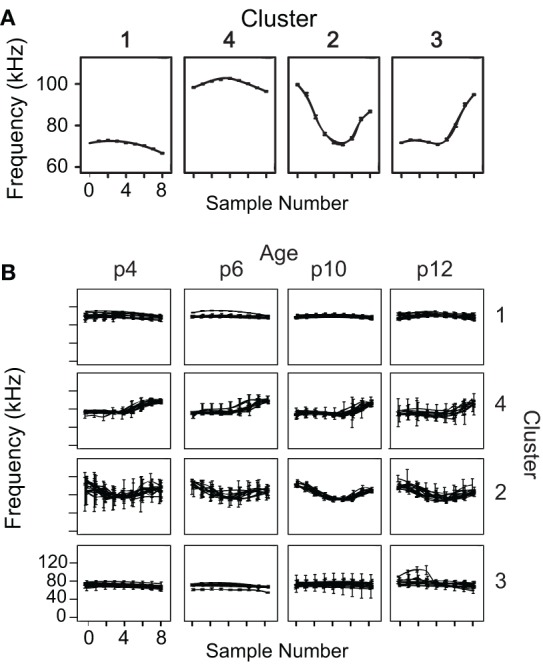
**Average frequency contours of the four identified syllable types. (A)** Average nine-point frequency contours, with 95% confidence intervals, are shown for the four syllable clusters determined by the two-step clustering algorithm. **(B)** The average frequency contours for each animal at each age are overlaid, demonstrating a high degree of overlap among animals that is robust across development.

Two-step cluster analysis was computed using four variables for each syllable: the total number of frequency steps and the start, middle, and end frequencies. Nested ANOVAs showed that the variables used within the cluster analysis did not show litter effects. There is significant variation among pups within litters [start frequency: *F*_(10, 4547)_ = 40, *p* < 0.001; center frequency: *F*_(10, 4547)_ = 21.6, *p* < 0.001; end frequency: *F*_(10, 4547)_ = 28.9, *p* < 0.001; number of frequency steps: *F*_(10, 4547)_ = 7.1, *p* < 0.001] and not significant variation among litters [start frequency: *F*_(2, 10)_ = 1.4, *p* = 0.298; center frequency: *F*_(2, 10)_ = 1.4, *p* = 0.302; end frequency: *F*_(2, 10)_ = 0.6, *p* = 0.561; number of frequency steps: *F*_(2, 10)_ = 2.8, *p* = 0.107]. Cluster analysis identified four clusters with an average silhouette of cohesion value >0.5. The average frequency contours of the four syllable types identified by the two-step cluster analysis are shown in Figure 4A. The average spectrograms of each pup are overlaid at each age to demonstrate that these four syllable categories are robust and highly overlapping across animals and development (Figure 4B).

**Figure 5:**
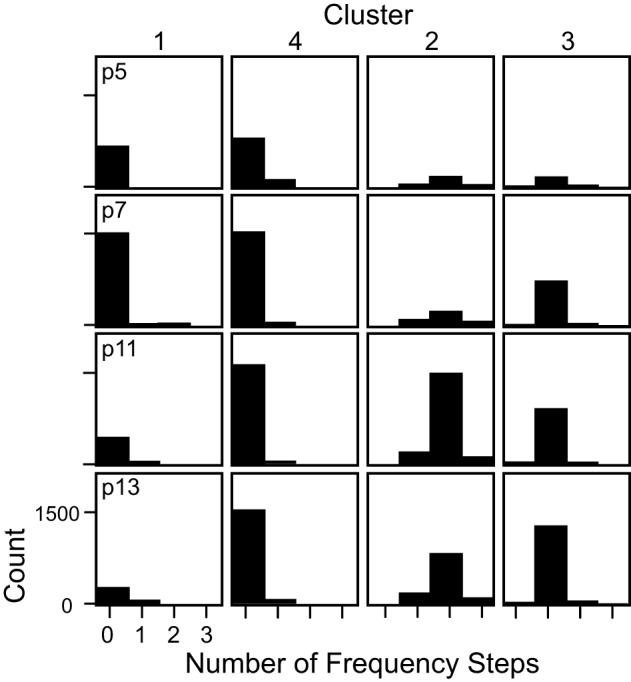
**The number of frequency steps contained within syllables in each cluster shown separately for each age.** Syllables in c1 and c4 typically had no frequency steps, syllables in c2 typically had 2 frequency steps, and syllables in c3 typically had only 1 frequency step.

**Figure 6:**
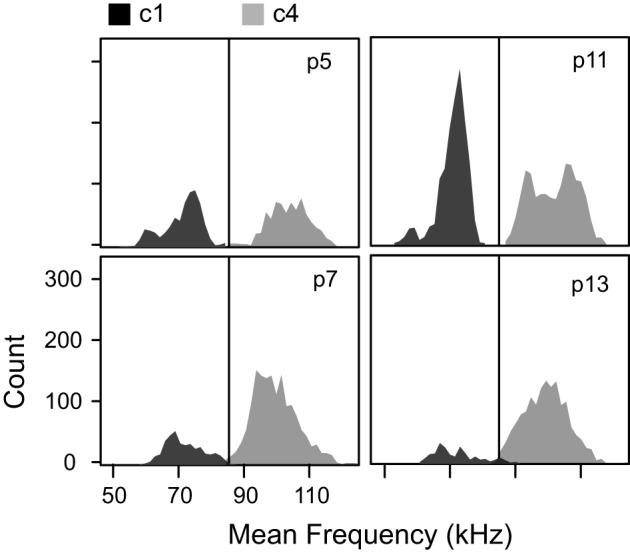
**Mean frequencies of the two clusters without frequency steps at each age.** The low frequency cluster (c1) and the high frequency cluster (c4) had distinct distributions of mean frequency. The *black vertical line* at 85 kHz marks the boundary set for the *mouse syllable classification calculator*.

Syllables in c1 and c4 were relatively narrow bandwidth signals that rarely contained frequency steps (Figure 5), a finding that was consistent across ages. Although c1 and c4 syllables share a very similar average frequency contour (Figure 4A), they differ dramatically in their frequency bands. The distributions of average frequency of syllables in these clusters differed dramatically (Figure 6). The bimodal frequency separability between c1 and c4 syllables is consistent across all ages. By examining syllables without frequency steps, it is clear that the mean frequency of syllables is not normally distributed at any age (Kolmogorov-Smirnov test *p* < 0.001), and a test of the coefficient of bimodality indicated that the distributions were significantly bimodal across ages (*b* > 0.55 for all ages). Figure 6 shows the separation in the bimodal distribution where the two-step cluster analysis split these syllables into their respective clusters, c1 and c4.

The majority of syllables in c3 had only 1 frequency step. The average frequency contour of this category (Figure 4A) starts low and then transitions to a higher frequency toward the end of the syllable. In contrast, c2 was comprised almost entirely of syllables with 2 frequency steps. To ensure that the inclusion of the number of frequency steps as a variable in the cluster analysis did not excessively overpower cluster classification, we removed the frequency step input variable from the clustering algorithm and re-computed the clusters. Four very similar clusters were characterized, with only 5% of syllables changing cluster, and the overall shapes of the averaged frequency contours did not change (data not shown). Syllables that changed cluster were predominantly those with frequency steps.

Interestingly, a One-Way ANOVA revealed a main effect of cluster on the syllable duration at each age [p5: *F*_(3, 3145)_ = 284, *p* < 0.001; p7: *F*_(3, 4329)_ = 369, *p* < 0.001; p11: *F*_(3, 4560)_ = 811, *p* < 0.001; p13: *F*_(3, 3082)_ = 384, *p* < 0.001]. At age p5 and p7, syllables in the high frequency cluster, c4, were significantly shorter than those in the lower frequency cluster, c1. However, they were significantly longer at p11 and p13 (Scheffe *post-hoc* correction, *p* < 0.001 for all comparisons). This finding was not evident in our previous manual characterization because syllables sharing similar frequency contours were pooled regardless of frequency band.

### Comparison with manual classification

**Figure 7:**
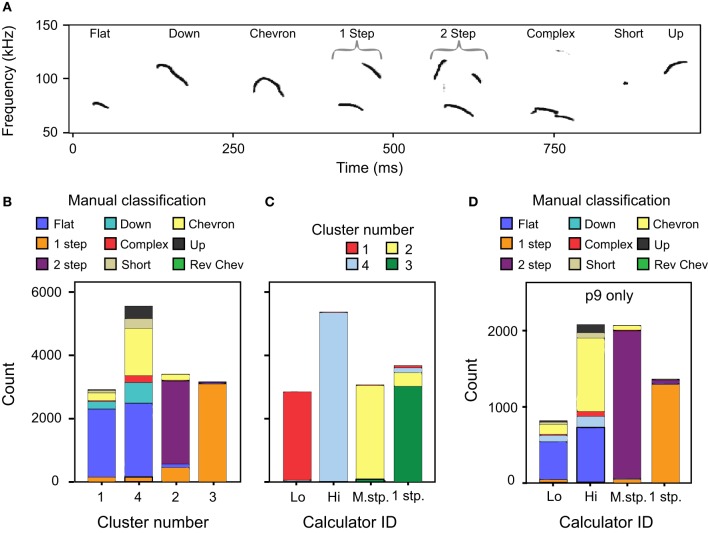
**A comparison of classification methods. (A)** Spectrogram of typical examples of p4 syllables defined using manual classification (Grimsley et al., 2011). **(B)** Comparison between the cluster analysis and manual classifications across age. Manual classification of 1 frequency step and 2 frequency stepped syllables match well to the cluster analysis. However, manually classified syllable types with no frequency steps are split between c1 and c4. **(C)** Comparison between cluster analysis classification and the *mouse syllable classification calculator* for all ages except p9. **(D)** Comparison between manual classification and the *mouse syllable classification calculator* for a novel data set of calls emitted by p9 pups. These syllables were not used in the two-step cluster analysis that defined the number of syllable categories.

The automatic classification of mouse pup syllables resulted in fewer distinct syllable types than our previous manual classification (Grimsley et al., 2011). Figure 7A illustrates the eight typical exemplars of syllables produced by p5 mouse pups and follows the nomenclature used in manual classification. Figure 7B shows the correspondence between manual classification and the cluster analysis classification.

#### Clusters 1 and 4

Clusters 1 and 4 include the vast majority of manually classified pup syllables that do not include frequency steps: flat, down, chevron, complex, short, and up syllables (Figure 7B). Although the manual classification of these syllable categories revealed two distinct frequency bands, a lower band between 60 and 80 kHz and a higher band between 90 and 120 kHz (Grimsley et al., 2011), categories were not based on the frequency band. In contrast, the two-step clustering algorithm groups these syllables depending on the frequency band they occupy. We explored why fewer syllable types were distinguished by cluster analysis than manual classification. We found that at each age the distribution of FM for manually classified up, down, and flat syllables (Figure 9A) did not reveal statistically justified borders that could serve as cut-off points for distinguishing among these syllable types. Although some of the distributions were not normal (Kolmogorov-Smirnov test *p* < 0.001), a test of the coefficient of bimodality indicated that those distributions were not significantly multimodal at any age (*b* < 0.3 for all instances). Further, there were no discrete peaks in the bandwidth distributions of the flat and chevron calls at any age (Figure 9B). We also conducted these analyses for individual pups within each age; none of the distributions were multimodal (*b* < 0.3 for all instances).

We found that cluster categories 1 and 2 could not be further separated into the manually classified syllable categories if other variables were used, or if frequency stepped syllables were removed from the analysis (data not shown). We also tried normalizing the frequency contour in the frequency dimension, as well as analyzing the data from different ages separately. The inclusion of all, or some combination of, the following additional variables did not result in cluster separation matching manual classifications and typically had minimal effect on the clustering: directional FM between the start and middle, directional FM between the middle and the end, bandwidth, all nine-point transitional FMs, syllable duration, mean dominate frequency, rate of FM between the start and the middle, rate of FM between the start and the end, the nine frequency contour points. Thus, although manual classifications tend to rely on arbitrary cutoff rules for distinguishing syllable types, the clustering algorithm shows that boundaries are neither distinct nor justified by a test of the coefficient of bimodality of the FMs of these syllables.

#### Cluster 2

Seventy-seven percent of the c2 syllables were manually classified as 2 frequency stepped syllables. Conversely, 98% of the manually classified 2 frequency stepped syllables were included in this cluster (Figure 7B). The other significant contributors to this cluster are syllables that had been manually characterized as 1 frequency stepped.

#### Cluster 3

Ninety-eight percent of the c3 syllables were manually classified as being 1 frequency stepped, and the majority (81%) of the 1 frequency stepped syllables were classified into c3 (Figure 7B). The remainder of the syllables classified into c3 were syllables that had been manually classified as 2 frequency stepped syllables.

**Figure 8:**
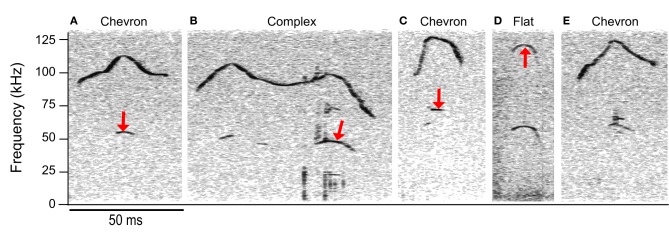
**Discrepancies between automated and manual analyses.** The manual classification is shown above each spectrogram. Syllables **(A–D)** were erroneously classified as having 2 frequency steps and were allocated to the c2 cluster. *Red arrows* indicate the points during each syllable where the automated extraction of the contour was not the same point on the spectrogram that the trained investigator would have chosen. Seventy-eight percent of the incorrect hits were in syllables with harmonic or non-linear components. Syllable **(E)** was correctly classified as having a continuous frequency contour even though there are non-linear components. Note that the lower frequency element in **(C)** is not the fundamental; this frequency has a value of 68 kHz, whereas a fundamental corresponding to the upper frequency component would be at 58.5 kHz.

Although cluster analysis distinguished fewer syllable types than manual classification, the syllable types computed were more acoustically discrete than those in manual classification. Some syllables manually classified as having no frequency steps were allocated into clusters principally comprised of frequency stepped syllables, e.g., clusters 2 and 3 (see Figure 7B). Figures 7A–D shows some examples of syllables that were manually characterized as having a continuous frequency contour, but were allocated into cluster 3, a cluster principally comprised of syllables with more than 1 frequency step. The fundamental of the syllables shifts suddenly in the examples shown in Figures 8A,B, however, the principal frequency contour is not discontinuous. This is not the case in Figure 8C, where the low and high frequency elements are not harmonically related. The example syllables shown in Figure 8 seem likely to be short-term release of a suppressed fundamental, though this cannot be confirmed here. Red arrows mark the points where the frequency contour measurements pick up either harmonic or non-linear elements within the syllable rather than the principal frequency contour that was identified by an investigator. The example shown in Figure 8E is very similar to those in Figures 8A,C, however, this syllable was correctly identified as having a continuous frequency contour. The majority of the discrepancies (78%) arose when syllables had non-linear or harmonic sub-elements. When syllables were produced at a low intensity, overlapped movement noise also generated erroneous transitions in the frequency contour.

### The mouse pup syllable classification calculator

In order to make these clusters more replicable for future classification of syllables, it was necessary to implement fixed categorical boundaries. To determine these boundaries, we explored the characteristics of each of the four clusters and used these values to generate a calculator in Microsoft Excel. This calculator requires the dominant frequency to be measured at nine evenly spaced points within a syllable. These values only need to be pasted into the calculator (Supplementary materials) and the syllable classifications will be determined automatically.

The syllable classification boundaries for the *mouse syllable classification calculator* are:

*The 1 frequency stepped syllable*. This syllable category corresponds closely to c3, which was almost, but not entirely, comprised of syllables with only 1 frequency step greater than 20 kHz (Figure 5). This syllable category includes any syllable that contains only 1 frequency step.
*The multi-frequency stepped syllable*. This syllable category corresponds closely to c2, which was almost, but not entirely, comprised of syllables with more than 1 frequency step (see Figure 5). This category includes any syllable that contains more than 1 frequency step.
*The low syllable*. This syllable category corresponds closely to c1. c1 syllables typically had no frequency steps (Figure 5), and had average frequency contours that were below 85 kHz (Figure 6). This category includes all syllables with no frequency steps, and mean frequency contours that are lower than 85 kHz.
*The high syllable*. This syllable category corresponds closely to c4. c4 syllables typically had no frequency steps (Figure 5) and had average frequency contours that were above 85 kHz (Figure 5). This category includes all syllables with no frequency steps, and mean frequency contours that are greater than or equal to 85 kHz.

The *mouse syllable classification calculator* was used to reclassify the CBA/CaJ syllables used for the cluster analysis. The classification of the two-step cluster analysis and the calculator correspond very well, with 94% coherence (Figure 7C). The differences in classification between the cluster analysis and the calculator are a result of the hard boundaries set for the calculator as opposed to the slightly softer ones used in the cluster analysis. We also compared the output of the calculator with manual categorization of data from p9 mice (*n* = 6306 syllables), a data set that was not included in the clustering algorithm. The calculator classification of syllables was highly consistent with manual classification for the 1 frequency stepped and the 2 frequency stepped syllables (Figure 7D) and took approximately 1 min to complete. The calculator classified 94% of the manually classified 1 frequency stepped syllables into the 1 frequency step category, and these syllables made up 95% of this category. Almost all (97%) of the syllables manually characterized as 2 frequency stepped were included in the multi-frequency stepped syllable group by the calculator, and these comprised 94% of this category. The remaining non-stepped syllables (manual classification categories: up, down, chevron, complex, flat, and short) were separated into the low and high category depending on their average dominant frequency.

The analysis method needs to be flexible enough to compensate for the different frequency space occupied by different mouse strains (Sales and Smith, 1978; Hahn et al., 1997; Wohr et al., 2008). To test how well the calculator generalized to other mouse strains we used two sample data sets from different strains. For both strains we compared manual classifications that were based on the four objectively-determined syllable categories with the result of the calculator's classification from IRW mice (*n* = 336 syllables) and C57BL/6 mice (*n* = 824 syllables).

The manual classification here follows the same fixed syllable classification boundaries informed by the cluster analysis. Prior to manual classification, the frequency thresholds used for the classification of syllables were examined for strain-dependent differences. The same criterion for a frequency step (20 kHz) was used for the IRW and C57BL/6 strains as was used to the CBA/CaJ syllables, as this also corresponded to the distributions of the frequency transitions in these strains (Figure 10A). However, for syllables with no frequency steps, the distribution of mean frequency differed between strains. Figure 10B shows that the 85 kHz threshold used to distinguish high and low frequency syllables in CBA/CaJ mice was not appropriate for IRW or C57BL/6 mice. The criteria for the high and low frequency syllables were thus shifted for the different strains. To account for strain-dependent differences in the frequency distributions, histograms are automatically drawn in the calculator and the threshold cut-off can be changed for each strain by the user. For both manual classification and the calculator, a criterion of 73 kHz was used to delineate high and low frequency syllables for IRW mice, and 70 kHz for C57BL/6 mice. Both mouse strains emitted syllables within the four syllable categories (Figure 10C). The calculator showed a 91% coherence with manual classification for IRW mice, and a 93% coherence for C57BL/6 mice.

## Discussion

Here we use a large data set of pup isolation calls to assess an objective syllable classification scheme based on cluster analysis and to compare with a previously published scheme based on manual classification (Grimsley et al., 2011). The spectro-temporal characteristics of these syllable clusters are then used to derive values that inform clear categorical boundaries for use in a Microsoft Excel-based calculator that automatically and rapidly classifies syllables in new data sets. This relatively straightforward method works well for classification of mouse pup isolation calls. The syllable categories are fewer than in previous reports based on manual analyses, but are more objective, well-justified statistically, and represent the spectro-temporal features of syllables. The *mouse syllable classification calculator* allows for the rapid and automated classification of syllables into call types using two commonly used software programs (SASLab, Avisoft Bioacoustics, and Microsoft Excel).

The isolation vocalization of pups are commonly used to assess disease state (D'Amato et al., 2005; Ricceri et al., 2007; Scattoni et al., 2008; Young et al., 2010; Wohr et al., 2011b) or stress level (Rupniak et al., 2000). Disease state in mice has been shown to change not only the rate of calling, but also the types of calls produced (Scattoni et al., 2008). For example, the proportion of the frequency stepped calls is elevated in a mouse tubular sclerosis model of autism (Young et al., 2010). In the same study, the proportion of low and high frequency calls differs even though the proportion of non-stepped calls does not change. These non-stepped calls correspond to the low and high syllables identified in our classification. The proportion of frequency stepped calls carries social contextual information in adult mice (Chabout et al., 2012). Thus, there is substantial value in objective tools that can evaluate calls in a broad range of mouse model systems.

The *mouse syllable classification calculator* allows for the rapid and robust classification of mouse pup isolation calls into syllable types. Experimenters only need to enter their data and the syllables will be classified automatically. This will allow experimenters to easily investigate differences in the probability of different syllable types being produced or to test for within-syllable changes that could reflect disease state or carry contextual information. Although our previous manual characterization of the mouse pup vocalizations took many weeks to complete, we were able to reclassify the same data set using this method in only a few hours. This method makes analysis of large data sets more feasible and cost effective. Automated classification of calls removes experimenter bias and allows for repeatability and comparability. However, the necessity for different frequency category thresholds between strains still renders inter-strain comparisons more complex. Even so, it allows the reader to better understand the features used in the classification, as it is not always clear what features are used in subjective categorizations and how distinct these features are. While other automated call classification methods have been described, such as hidden Markov models (Ren et al., 2009) and neural network models (Pozzi et al., 2010), these depend on pre-determined categories.

We took an alternate approach by first computing the number of acoustically distinct syllables using a two-step clustering algorithm and using the boundaries of these clusters to classify syllables. As input variables for the two-step clustering algorithm, we used spectro-temporal features of the syllable that experimenters report using when distinguishing among syllables by manual classification (Berryman, 1976; Portfors, 2007; Scattoni et al., 2008; Grimsley et al., 2011; Sugimoto et al., 2011).

The four parameters used in the clustering algorithm were chosen in an attempt to replicate those used in manual classifications of the calls, in which the shape of the frequency contour is the principal factor used. The easiest variable to characterize by eye, and in automated analysis, is the presence or absence of a discontinuous element in the contour, a frequency step. We used a nine-point contour to measure for frequency steps; however, we used only the start, middle, and end frequency for the clustering algorithm. The rational for using a simplified contour for the clustering algorithm was due to an issue that arose in our initial analysis of these data. When using many points within the frequency contour as variables within the clustering algorithm, the measurement point where a frequency step occurred outweighed the other variables enormously. The result was that many clusters were formed, one for each sampling point where a frequency step could occur; point 5, 6, 7, 8, and so on. By slimming down the analysis to measure the number of steps, then recording the start, middle, and end frequencies, the cluster algorithm better matched manual classification, and the presence of a frequency step was not overpowering the analysis.

**Figure 9:**
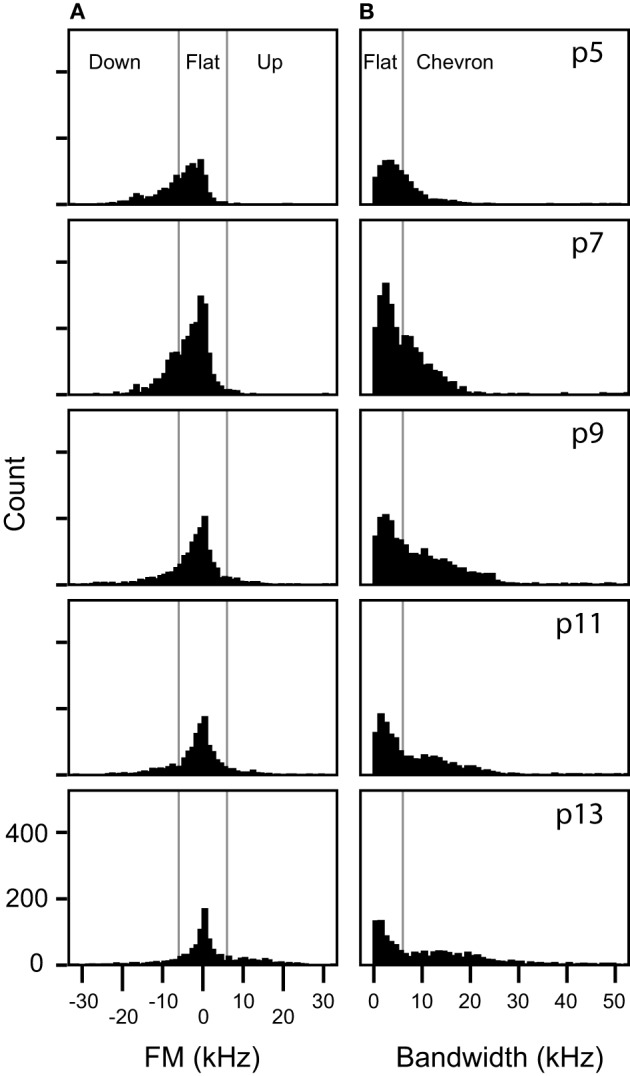
**Frequency modulations are distributed continuously in non-stepped syllables. (A)** Distributions of the frequency modulation of syllables manually classified as up FM, flat, or down FM. The vertical lines mark the 6 kHz of FM boundaries that was arbitrarily set for manual classification for these syllables. **(B)** Distributions of bandwidth of syllables manually classified as flat or chevron. The vertical line marks the 6 kHz boundary that was arbitrarily set for manually distinguishing between flat and chevron syllables.

The use of the start, middle, and end frequencies of syllables would separate the up FM, down FM, flat, and chevron syllables if they are acoustically distinct. A chevron-type cluster would comprise syllables in which the start and end frequencies are always lower than the center frequency. An up FM cluster could be formed if, for a distinct population of syllables, the center frequency is higher than the start frequency and the end frequency is higher than both the start and center frequency. As Figure 9 displays, there are no sub-populations in the probability distributions at each age in the bandwidth of flat and chevron calls, nor are there sub-populations within the directional bandwidth of the up FM, down FM, and flat calls.

**Figure 10:**
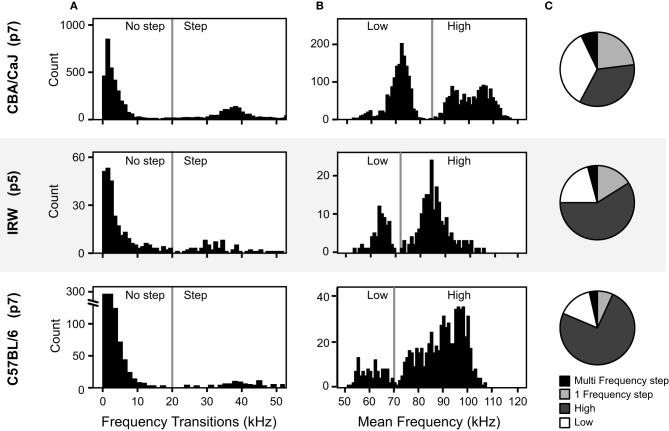
**Comparison of acoustic features of pup vocalizations in three mouse strains. (A)** Distributions of the frequency transitions. The vertical lines at 20 kHz marks the criterion for a frequency step across the three strains. **(B)** Distributions of the average frequency of syllables without frequency steps. The vertical lines mark the boundary between high and low syllables, which differs among strains. **(C)** The proportion of each syllable type for the three mouse strains, based on the *mouse syllable classification calculator.*

Hammerschmidt et al. (2012) analyzed mouse pup syllables using two-step cluster analysis and were also unable to separate the FM syllables into distinct categories. Their analysis characterized only two clusters, short syllables and long syllables. They used eight acoustic variables for the cluster analysis; start frequency, peak frequency, maximum FM change in 0.21 ms, location of the maximum frequency, location of the peak frequency, duration, and duration of amplitude gaps within a syllable. The introduction of factors such as the duration of the amplitude gaps could introduced noise into the clustering algorithm, since small movements in the head of a vocalizing pup can affect the amplitude of signals and generate variables that are not present at the mouth. Furthermore, it is not clear how many syllables were included in their two-step cluster analysis. If too few syllables were included, the cluster analysis that is designed for thousands of data samples would be underpowered, resulting in the identification of too few clusters. Hierarchical cluster analysis is more appropriate under conditions of smaller sample sizes.

Our previous manual classification of mouse syllables across development determined eight ultrasonic syllable types at age p5 (Grimsley et al., 2011) based on the syllable categories set out by Scattoni et al. (2008). However, the automated analysis only determined four distinct syllable categories. The discrepancies between manual and automatic classifications were primarily evident among syllables that do not contain frequency steps. Thus, the five manually classified syllable types with no frequency jumps (flat, up FM, down FM, short, and chevron) were automatically clustered into only two categories, based on the frequency band they occupied rather than on the shape of the spectrographic contour. The distinct frequency bands in pup syllables were reported previously (Liu et al., 2003) and were identified in our published, manually-based analysis (Grimsley et al., 2011). It appears that the larger number of syllable types identified in the manual classifications is based on trained investigators' abilities to form categorical, albeit arbitrary, distinctions relating to frequency transitions. However, we show here that there is a continuum of frequency transitions and an analysis of the probability distributions does not allow clear borders to be identified. As a result, the clustering algorithm did not identify these as distinct syllable types.

The results of this study raise fundamental questions regarding syllable classification. For example, at what point is a syllable sufficiently frequency modulated to be considered an up FM syllable? Our review of the literature indicates that the FM borders in analyses of mouse syllables have not been based on a separation in the probability distributions of the directional FM of the calls. Instead, the identification of separate categories of up FM, down FM, and flat calls is the result of an arbitrary segmentation of a continuous distribution. Unless behavioral studies show that mice can form such perceptual categories (see below), there is no justification for the categories. Does the reduced number of categories from this analysis hamper detection of altered repertoires due to strain differences or pathological processes? Our view is that the statistically based analysis of changes in robust, non-arbitrary categories will lead to a more effective assessment of changes in vocal repertoire.

A caveat to this assessment is the recognition that variability within the non-stepped syllables, identified by manually-based analyses, may nonetheless be perceptually distinct to mice. In human speech sounds, the English phonemes /b/, /d/, and /g/ are produced along an acoustic continuum, yet comprehension of these sounds is split into three distinct phonemes with hard borders in a phenomenon termed categorical perception (Liberman et al., 1957). There is evidence for categorical perception of frequency modulated stimuli in the Mongolian gerbil (Wetzel et al., 1998) and the rat (Mercado et al., 2005). Further behavioral experiments would be needed to test if mice use categorical perception to discriminate among the non-stepped syllables based on the categorical features of their frequency transitions. If so, the psychophysical boundaries could be used to further segregate these syllables.

## Conclusions

A two-step cluster analysis identified four acoustically discrete syllable types produced by mouse pups. Psychophysical analysis is required to determine whether mice can acoustically distinguish among these syllables, or if they can categorically distinguish subtypes within the continuum of frequency transitions found in the non-frequency stepped syllables. We used the boundaries of the syllable clusters to generate a tool within Microsoft Excel for the automatic classification of mouse syllables using a nine-point measurement for the dominant frequency. Although there are strain or recording context dependent differences in some features of isolation calls such as; the frequency space that calls occupy, the duration (Sales and Smith, 1978; Hahn et al., 1997), and the proportion of each of the syllable types produced (Branchi et al., 1998), we demonstrated that isolation calls from different mouse strains can be analyzed using this method by shifting the frequency boundaries of the clusters to those of the novel strains. This tool can be used by behavioral neuroscientists to investigate the relationship between mouse pup vocal signals and genetic makeup, health status, or emotional state.

### Conflict of interest statement

This research was financially supported by NIH R01 DC00937 and DC00937-19S1.
